# Studying Individual Differences in Language Comprehension: The Challenges of Item-Level Variability and Well-Matched Control Conditions

**DOI:** 10.5334/joc.317

**Published:** 2023-09-07

**Authors:** Lena M. Blott, Anna E. Gowenlock, Rogier Kievit, Kate Nation, Jennifer M. Rodd

**Affiliations:** 1 Department of Experimental Psychology, University College London, UK; 2 Cognitive Neuroscience Department, Donders Institute for Brain, Cognition and Behavior, Radboud University Medical Center, Nijmegen, The Netherlands; 3 Department of Experimental Psychology, University of Oxford, UK

**Keywords:** Individual Differences, Language Comprehension, Lexical Ambiguity, Semantic Ambiguity, Sentence Comprehension

## Abstract

Translating experimental tasks that were designed to investigate differences between conditions at the group-level into valid and reliable instruments to measure individual differences in cognitive skills is challenging (Hedge et al., 2018; Rouder et al., 2019; Rouder & Haaf, 2019). For psycholinguists, the additional complexities associated with selecting or constructing language stimuli, and the need for appropriate well-matched baseline conditions make this endeavour particularly complex. In a typical experiment, a process-of-interest (e.g. ambiguity resolution) is targeted by contrasting performance in an experimental condition with performance in a well-matched control condition. In many cases, careful between-condition matching precludes the same participant from encountering all stimulus items. Unfortunately, solutions that work for group-level research (e.g. constructing counterbalanced experiment versions) are inappropriate for individual-differences designs. As a case study, we report an ambiguity resolution experiment that illustrates the steps that researchers can take to address this issue and assess whether their measurement instrument is both valid and reliable. On the basis of our findings, we caution against the widespread approach of using datasets from group-level studies to also answer important questions about individual differences.

## Introduction

### Studying Individual Differences in Language Skills

Psycholinguists have become increasingly interested in studying individual differences in language processing. Such individual differences exist across the linguistic domain, from vocabulary knowledge and syntactic processing to discourse comprehension, and they manifest at the behavioural and neurological level (see Kidd et al., 2018, for an overview). Some children achieve language processing milestones at an earlier age, and/or have consistently higher performance across a range of language ability domains. Such individual differences can be surprisingly stable across the lifespan (e.g. McNeish, Dumas, & Grimm, 2020). Moreover, individual differences in cognitive ability, including language ability, predict a range of outcomes including educational attainment, job success and even morbidity and mortality (e.g., Deary, Weiss, & Batty, 2010; Johnson, Beitchman & Brownlie, 2010; Der, Batty, & Deary, 2009). Understanding sources of variation in language skills and their relationship with other cognitive domains is paramount for building powerful theories of language development and processing, and to develop interventions that target language outcomes.

To study individual differences, researchers need instruments that measure performance adequately and can reliably capture variation between individuals. Psycholinguists may first look to the clinical and educational language sciences for standardised assessments that have been normed on representative samples (e.g., Test for the Reception of Grammar, Bishop, 1983; the Woodcock-Johnson battery, Woodcock-Johnson, 1977; Clinical Evaluation of Language Fundamentals (CELF), Semel, Wiig, & Secord, 2013). Such assessments tend to capture language skills in a relatively broad sense, by scoring definitions and answers to comprehension questions for example. While this approach can be highly effective in rank ordering individuals, the contribution(s) of specific cognitive components to these composite measures can be difficult to disentangle. From a psycholinguistic perspective, we often wish to understand performance on language tasks in terms of variation in underlying constructs or processes. Even for a straightforward task like picture-word or picture-sentence matching, a range of linguistic and cognitive factors are likely to influence performance (including e.g., the quality of lexical representations, activation of relevant background knowledge, inhibition of irrelevant information, processing speed, working memory, and inference making). Generally, standardised assessments are not well suited to capturing variability across the population in skills that relate to a particular sub-domain of language or a specific linguistic phenomenon.

Psycholinguists have therefore needed to create bespoke tests that target specific language skills for use in a single experiment or set of experiments within their own laboratory. For example, research into individual differences in ambiguity resolution has used measures as varied as reading time (self-paced or observed via an eye-tracker, e.g. Daneman & Carpenter, 1983; MacDonald, Just, & Carpenter, 1992; Kemper, Crow, & Kemtes, 2004; Evans et al., 2015; Blott, Rodd, Ferreira, & Warren, 2020), electrophysiological event-related potentials (e.g. Lee & Federmeier, 2011), comprehension question accuracy (e.g. Engelhardt, Nigg, & Ferreira, 2017), interference from irrelevant meanings in a semantic priming paradigm (e.g. Gernsbacher, Varner, & Faust, 1990), accuracy and response times of sentence-level judgements about meaningfulness (e.g. Gernsbacher, Robertson, & Werner, 2001) or grammaticality (e.g. Vuong & Martin, 2014), single-word naming within sentence contexts (e.g. Hopkins, Kellas, & Paul, 1995; Dagerman, MacDonald, & Harm, 2006) and sentence-picture matching (e.g. Malyutina & den Ouden, 2016).

Developing bespoke tasks to capture individual differences raises numerous challenges. It requires defining the construct-of-interest (what do we want to measure?) and operationalising it (how should we measure it?), and considering the validity and reliability of the measurement (does our task measure what we want to measure and does the outcome measure rank-order individuals appropriately?) (Goodhew & Edwards, 2019; Hedge et al., 2018; Tucker-Drob, 2011). Our aim is to explore some of these challenges associated with using an experimental approach to capture individual differences in language processing abilities. Our focus is on subtractive designs. These designs rely on the comparison or “subtraction” between different experimental conditions to isolate a particular aspect of linguistic processing. We present the data from a task designed to measure individual differences in adults’ ability to resolve lexical ambiguities as a case study to illustrate these challenges, and make recommendations that may assist in developing and evaluating other studies of individual differences in language processing.

#### Using subtractive designs for individual differences research

Many group-level experiments compare performance in a critical condition of interest to a carefully constructed control/baseline condition. The rationale of this approach is that general factors associated with task performance are controlled such that any difference across conditions can be attributed to the experimental manipulation. Consider for example a classic semantic priming experiment. In the critical primed condition participants will make lexical decisions (i.e. decide if letter strings correspond to real words) to target words that are preceded by semantically related primes (e.g., cat-DOG). This experimental condition is typically compared to a baseline condition where the *same* target words are preceded by closely matched unrelated primes (e.g., car-DOG). Similarly, an experiment might attempt to isolate the linguistic process of lexical ambiguity resolution by comparing task performance on sentences containing ambiguous words (e.g., “organ”) to performance on identical sentence frames where the target word is replaced by an appropriate unambiguous control word (e.g., “The expert knew that the damaged organ/piano would be quite difficult to tune”; Blott et al., 2022). (Note that we use ‘unambiguous’ here, and throughout the manuscript, to mean words that are relatively low in ambiguity while recognising that very few words are truly unambiguous in the sense of having only one specific referent.) This approach allows us to hold constant many of the linguistic and cognitive processes that are necessary components of the task (e.g., letter identification, word-form processing, grammatical parsing, response selection and button pressing) and to subtract these out to isolate the underlying process of interest (cf. Donders, 1868 [1969]).

Although many influential and well-replicated findings have emerged from group-level experiments that use this approach to stimulus matching, we cannot assume that the same tasks which reliably demonstrate a (linguistic) effect of interest are also reliable in their ability to rank-order individuals (Hedge et al., 2018; Rouder et al., 2019; Rouder & Haaf, 2019). Task reliability is vital for individual differences research, as correlations between measures are attenuated when the measures themselves are not reliable (Hedge et al., 2018). Looking at the reliability of a number of subtractive tasks that show robust group-level effects, Hedge and colleagues (2018) reported poor test-retest reliability for such classic cognitive tasks as Stop-signal, Stroop, Eriksen flanker, Posner cuing, and Navon. Indeed, some tasks may well have become popular for group-level designs precisely *because* of their relatively small between-participant variability, which makes them well powered to consistently detect group-level differences but unsuitable for individual differences research (Goodhew & Edwards, 2019; Enkavi et al., 2019; Schuch et al., 2021; Byers-Heinlein et al., 2022). It remains to be seen whether a similar pattern emerges for well-established psycholinguistic tasks.

It is likely that the low reliability observed in the cognitive tasks mentioned above is partly due to the scoring method by which performance was captured, rather than reflecting an intrinsic limitation of the tasks themselves (see Hedge et al., 2018). A prevalent approach in subtractive designs is to calculate difference scores, simply the numerical difference in participants’ mean performance across two conditions (see e.g., Rogosa & Willett, 1983). As has long been recognised in the literature on the measurement of change, however, “the difference between two fallible measures is frequently much more fallible than either” (Lord, 1963, p. 32). Difference scores have several disadvantages. Firstly, aggregating task performance in each condition to calculate a difference reduces the *between*-participant variability that we are interested in (Hedge et al., 2018). Secondly, aggregate scores for each individual contain trial-by-trial nuisance variation, meaning that these scores are notoriously noisy and unreliable estimates of individual effect sizes. Thirdly, the reliability of difference scores critically depends on the number of trials the participant has completed (Rouder & Haaf, 2019), which may vary across participants due to data cleaning/exclusions. Fortunately, statistical approaches are available that allow us to derive more reliable individual scores from condition comparisons, avoiding the need to rely on difference scores. Rouder and Haaf (2019) recommended the use of hierarchical modelling using trial-level data to derive estimates of underlying effect sizes for individual participants, while others have discussed the benefits of diffusion models in estimating traits using response time measurements (e.g. Schubert et al., 2016; Ratcliff & Childers, 2015). While these innovative statistical approaches resolve some of the issues with using subtractive designs for individual differences research, they cannot overcome some of the specific challenges of translating the experimental designs commonly used by psycholinguists into individual difference measures.

#### What’s so special about language? The problem with multi-version experiments

In language experiments, the problems associated with subtractive designs for individual differences research are compounded by the nature of the stimuli. Linguistic stimuli are typically complex and vary along numerous dimensions that can be hard to quantify, but that can affect task performance. Even for single words, processing is influenced by factors such as frequency, age of acquisition, word length, phonological/orthographic neighbourhood density, and word class (e.g., Mandera et al., 2020). With multi-word stimuli such as phrases, sentences or paragraphs, things get even more complex. To some extent, the variability inherent in linguistic stimuli can be dealt with at the analysis stage by treating ‘item’ as a random effect so that findings can be generalised to other non-tested linguistic stimuli – an approach that is well-established in psycholinguistics. For example, experiments typically include both by-subject and *by-item* analyses (Clark, 1973), or capture effects of item variability in mixed effects analyses (Baayen et al., 2008). Importantly, item variability must also be addressed when first designing an experiment. In particular, for subtractive designs, it is important that while conditions differ in the process of interest, they differ as little as possible on other extraneous variables. As described above, this often results in re-using the same or very similar linguistic items across different stimulus conditions. Although this approach has clear benefits in terms of stimulus control, an unfortunate consequence is that it is often inappropriate to present both variants of a matched stimulus pair to any given participant, at least within the same experimental session. Participants will likely remember items that they have encountered before, and there is robust evidence for repetition priming and practice effects that make repetition of (parts of) linguistic stimuli highly problematic (Forbach et al., 1974; Forster & Davis, 1984; Ledoux et al., 2006; Rodd et al., 2013; Stark & McClelland, 2000). Although it may be possible to capture such priming/practice effects, at least to some extent, within our analysis models, the presence of such repetition may result in qualitative changes in participants’ performance (see Maciejewski et al., 2020, for a demonstration of how priming effects can reduce the effect of interest in later trials). Even with a reasonably long interval between encounters, psycholinguists typically avoid presenting the same (or very similar) material to the same person twice, lest the repetition affects their processing (see Cave et al., 1997, for evidence of repetition priming effects that persist after 48 weeks). As a result, psycholinguists routinely use multi-version designs in their group-level experiments, with different groups of participants being randomly assigned to different experimental versions (or lists) that each contain different subsets of the experimental materials. This approach ensures that while each participant contributes data to all experimental conditions, they each only see one version of any matched pairs of items and that, across participants, all items contribute to all experimental conditions.

This type of multi-version design has two adverse consequences for researchers aiming to translate group-level experiments into individual differences measures. First, it reduces the number of items that can be presented to any given participant. For example, in the case of a two-condition subtractive design, the number of stimuli presented to each participant will be halved. Fewer trials mean an increase in measurement error, and reduced measurement reliability (Rouder et al., 2019). Second, in multi-version experiments different groups of participants will encounter different sets of stimuli, introducing an additional source of between-participant variance, which is not present in single-version experiments. Instead, observed between-participant differences could be driven by differences between the experimental versions in overall task difficulty or in the sensitivity to between-condition differences. Although between-versions differences can potentially be dealt with at the analysis stage, there is little (if any) benefit to using multi-version designs in individual differences research, making it hard to justify introducing this additional complexity.

Of course, abandoning the multiple-version experiment for individual differences studies creates new difficulties for psycholinguists. As described above, if we present all stimuli to all participants, we cannot include any matched pairs of stimuli that are sufficiently similar, as they are likely to produce within-pair priming or practice effects. At the same time, conditions should be matched as closely as possible except for our factor-of-interest (e.g. ambiguity), so that we can make valid inferences about individual differences in our construct-of-interest (e.g. disambiguation ability).

Here, we present as a case study an adapted multi-version, multi-condition psycholinguistic experiment and consider its utility for addressing questions about individual differences. Our aim was to develop a task that would allow us to detect reliable individual differences in the ability to use contextual cues for disambiguation during online spoken discourse processing. We discuss the challenges we faced, assess the solutions we proposed, and make recommendations for future research.

### Case study: An individual differences paradigm to measure disambiguation skill

Successful comprehension of written or spoken language, requires the meanings of individual words to be activated and integrated into the wider discourse context such that a coherent mental model of the input can be constructed (Graesser et al., 1994, 1997; Van Dijk & Kintsch, 1983). Given that most English word forms are associated with multiple meanings (Rodd et al., 2002; Rodd, 2018, 2022), cues from the surrounding context must be used to activate and integrate the *context-relevant* meaning representation of each word (Duffy et al., 1988; Eddington & Tokowicz, 2015; Rodd, 2020). For example, the word “positive” is often used to describe a desirable event or feeling, but when awaiting a clinical test result, the same word form can take on the opposite meaning. Previous research suggests that there is substantial variability in the efficacy with which individuals detect and use cues in the surrounding context to aid online lexical processing (e.g. Federmeier & Kutas, 2005; Gernsbacher, 1993; Khanna & Boland, 2010; Lee & Federmeier, 2011; Nation & Snowling, 1998; Norbury, 2005; Snedeker & Trueswell, 2004; Trueswell et al., 1999).

We developed a novel paradigm to measure participants’ ability to successfully resolve lexical-semantic ambiguities as they listen to short narratives. We used auditory presentation and used responses to picture probes as our outcome measure so that the task could be portable across different participant populations (e.g. children, older adults, individuals with language impairments), and could measure disambiguation skill independently from variation in reading ability. The experiment was accessed remotely on the Gorilla experiment platform (www.gorilla.sc/about; Cauldron Inc.; Anwyl-Irvine et al., 2019), allowing recruitment from larger and more diverse groups of participants, who may be less likely to attend lab-based testing sessions.

**Figure 1:**
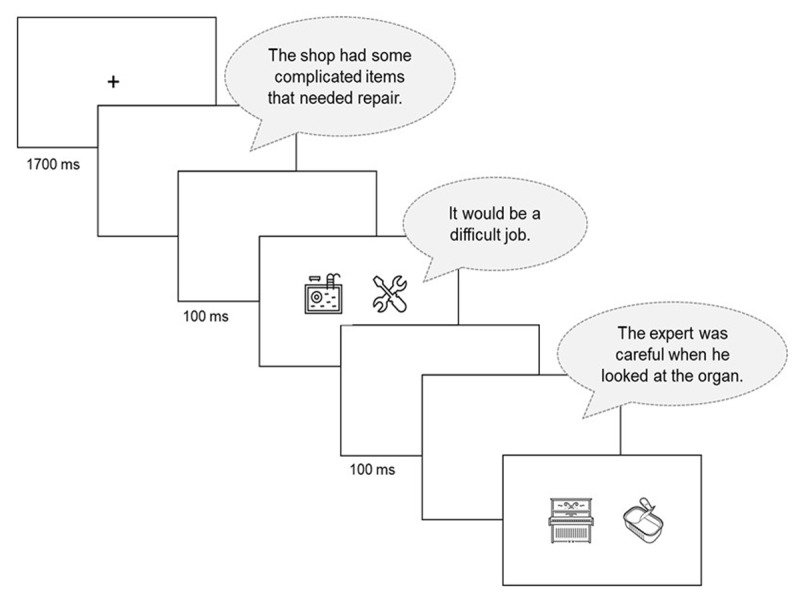
Illustration of the trial structure of a single Ambiguous trial in the picture selection task. Each trial comprised a 3-sentence auditory narrative. Participants were instructed that whenever pictures appeared on the screen they were to select the picture which “fits best with what [they had] just heard in the story”.

Several constraints were taken into account when designing the materials and task. Narratives in the Ambiguous condition were constructed to introduce a relatively challenging (but reasonably naturalistic) disambiguation situation in which participants must select an appropriate meaning of an ambiguous word (e.g., organ) on the basis of its preceding context. We chose to construct narratives in which disambiguating cues *precede* the ambiguous word since disambiguating cues which appear *after* the ambiguous word likely load on a range of other cognitive processes such as error detection and monitoring in addition to our construct-of-interest. For an illustration of the task, see Figure 1.

The Ambiguous condition comprised three-sentence auditory narratives in which the final word was ambiguous, and the preceding context was more consistent with its subordinate (low-frequency) meaning compared with its dominant (high-frequency) meaning (e.g., musical organ vs bodily organ, see Table 1). To enhance the cognitive challenge associated with disambiguation, we only included relatively weak linguistic disambiguation cues. Specifically, we set up a general scenario that led to the intended (subordinate) meaning being a ‘better fit’, while avoiding, as much as possible, any lexical primes that would strongly point towards the intended meaning (e.g., the context for “organ” avoided associates such as “music”, “keyboard” and “instrument”). Indeed, in some cases the constraint was sufficiently weak that there was a possible (albeit implausible) interpretation that could be consistent with the non-intended meaning. For example, in the example presented in Table 1, listeners must select the appropriate meaning for “organ” based on the contextual constraint that musical organs are more likely to be repaired in shops than are bodily organs. We included a filler sentence (Sentence 2) to introduce a temporal delay between the disambiguating information (Sentence 1) and the ambiguity (final word of Sentence 3) to increase working memory demands and thereby magnify any observed experimental difference between performance on these items and the unambiguous control items.

**Table 1:** Examples of 3-sentence narrative structures in the three conditions.


CONDITION	SENTENCE	EXAMPLE NARRATIVE

Ambiguous	Sentence 1	The shop had some complicated items that needed repair.

	Sentence 2	It would be a difficult job.

	Sentence 3	The expert was careful when he looked at the organ.

Unambiguous (item-matched)	Sentence 1	The shop had some complicated items that needed repair.

	Sentence 2	It would be a difficult job.

	Sentence 3	The expert was careful when he looked at the piano.

Unambiguous (set-matched)	Sentence 1	Gina pinned the piece of cotton onto the doll.

	Sentence 2	It didn’t seem right.

	Sentence 3	She thought it might look better with some leather.


The most unusual aspect of this experimental design is that it contains two baseline conditions. The first ‘Unambiguous (item-matched)’ condition follows the psycholinguistic conventions for standard group-level designs: it provides an item-wise matched unambiguous control by replacing the ambiguous target word (e.g., “organ”) with an unambiguous target that is sufficiently similar in meaning so that an identical narrative frame can be used across these two conditions. The same picture probes could also be used for both the Ambiguous and Unambiguous (item-matched) condition (i.e., picture of an object that was rated as both an acceptable “piano” and “organ”). This allowed us to measure, at the group level, the main effect of Ambiguity on task performance (i.e., responses to the picture probe in terms of accuracy, and response times on correct trials). Based on the ambiguity processing literature we predicted accuracy to be significantly lower and response times significantly longer for the Ambiguous compared to the Unambiguous (item-matched) condition, indicative of disambiguation-related processing costs (a classic “subordinate-bias effect”; Pacht & Rayner, 1993).

This closely item-wise matched control condition is however suboptimal for an individual differences design: it is inappropriate to present both variants of such closely matched stimulus pairs to the same participant. Thus, we also created an additional control condition, referred to throughout as the ‘Unambiguous (set-matched)’ condition (Table 1). Items in this condition are sufficiently different to the Ambiguous narratives to allow within-participant repetition. Inevitably, this approach brings with it a loss of control over item matching. The Unambiguous (set-matched) condition was not item-wise matched to the Ambiguous condition, meaning that the conditions differed not only in the ambiguity of the final word (targeting our construct-of-interest, disambiguation skill) but also in the preceding words and sentences. However, we attempted to match the Unambiguous (set-matched) condition to the Ambiguous condition as best we could across the entire set of items. We describe below the attempts we made to ensure that the unambiguous condition was as well-matched as possible on variables deemed most likely to influence task performance, but it was not possible to control fully. At the group level, we expected participants to show very similar performance in the two Unambiguous conditions relative to the Ambiguous condition, given that neither required disambiguation. By including both in our experiment, we were able to assess group performance for the Ambiguous condition against both types of Unambiguous control. This allowed us to establish the viability of the Unambiguous (set-matched) condition at a group-level before using it in our analysis of individual differences.

To measure ambiguity resolution on-line, we used a simple picture selection task. At the acoustic offset of the final word (which we refer to as the target word) in each narrative (e.g., “organ”, “piano” or “leather”; Table 1), two pictures appeared on the screen, one representing the intended meaning of the target and the other an unrelated meaning. Participants were instructed to select the picture that best fit the meaning of the narrative. Poorer picture selection performance in the Ambiguous condition relative to the Unambiguous conditions would be assumed to reflect the increased difficulty that participants experience in retrieving the contextually relevant meaning of the ambiguous word (Betts, 2018; Foss et al., 1968). Importantly, this paradigm avoided the need for expressive language, and for hand-scoring or transcribing participant responses (as would be the case for other tasks commonly used to assess participants processing of ambiguous words such as reading-aloud homographs, or providing definitions for words, e.g. Brock, Sukenik, & Friedman, 2017; MacGregor et al., 2020).

We used a 2-alternative forced-choice (2-AFC) picture-selection task over a yes/no decision to a single picture to reduce the impact of individual differences in response thresholds, particularly in accuracy data (Egan, 1975; Green & Swets, 1966). Decisions to respond in a single-picture yes/no task rely on criteria as to when to initiate a response (“Does this picture fit *well enough* into the narrative to warrant a ‘yes’ response?”). Such thresholds would likely vary across participants, and introduce a source of individual differences that we were not interested in in the present study. Instead, a 2AFC paradigm meant that participants’ decision on each trial was restricted to “Which of these pictures fits *better*?”, explicitly providing participants with a common response criterion. Finally, the irrelevant (dominant) meanings of the ambiguous words were *never* depicted during the task, as this would have potentially boosted the activation of these irrelevant word meanings, and artificially increased the load on processes such as conflict resolution and inhibition. The inclusion of irrelevant meanings might have also caused unnecessary confusion in some participants as to exactly what was required of them as such distractors could potentially be interpreted as being related to the narratives.

A potential concern with this task is that presenting a picture that relates to the target meaning could provide an additional disambiguation cue. Plausibly, for some trials at least, a participant may not have fully resolved the ambiguity when the pictures are presented, leaving open the possibility that the pictures may provide both general encouragement to participants to resolve the ambiguity, and specific semantic cues that aid in the disambiguation process. Our view is that for our current purposes this is not problematic – language in the real world is situated in context and visual cues may well contribute to ambiguity resolution in these circumstances. In our task, any variation in the extent to which participants are able to use these additional visual cues will only act to amplify individual differences in the disambiguation process, our focus of interest.

In summary, participants listened to multi-sentence narratives that ended in an ambiguous word (or unambiguous control word), and selected from two alternative picture probes the picture that best represented the content of the story. We present data from an initial experiment with 50 adults. Our aim was to assess whether this task can be used as a valid measure of disambiguation skill and test its reliability for future use as an individual differences instrument. Specifically, we aimed to:

1. Confirm the presence of a group-level ambiguity effect, using a well-matched control condition (Unambiguous (item-matched)) and a conventional multiple-version design.
2. Establish the appropriateness of a new control condition (Unambiguous (set-matched)) suitable for a within-participant assessment of ambiguity resolution ability.
3. Test whether reliable individual differences could be detected in the present sample.

## Method

### Participants

Participants were recruited via the online recruitment platform Prolific Academic (www.prolific.co). They were eligible if they were native speakers of English, currently residing in the United Kingdom and had spent most time before age 18 in the United Kingdom, were aged between 18 and 40, had no uncorrected visual or hearing impairments, no diagnoses of language difficulties, and had an approval rating of at least 80% on Prolific and had not participated in any pre-tests using these stimuli. These exclusion criteria were applied via the Prolific participant database.

Data from 50 participants (29 male, 21 female; 44 monolingual, 6 bilingual; mean age 30.0 (SD 6.92, range 18–41)) were included in the analysis. Thirty-seven additional participants were excluded prior to the experimental task due to failure of the audio technology check (n = 36), or because they indicated that they had an uncorrected visual/hearing impairment or diagnosed language deficit (n = 1). One additional participant reported technical difficulties with the display of picture probes and their data were excluded. We treated this data as a small-scale pilot study with the aim to validate the picture-selection task and conditions in a group-level design in the first instance. Given the novelty of the task and the associated uncertainty about the size of group-level effects of the ambiguity disadvantage, let alone the size of any potential individual differences in that effect size, an *a priori* power analysis was not feasible. The present study was therefore not preregistered, and the sample size was not based on any *a priori* calculations. We will return to this limitation in the Discussion. Informed consent was obtained from all participants and they were paid £3.75 each, with most people taking about 30 minutes for the study. The study was approved by UCL’s Department of Psychology and Language Sciences Ethics Chair.

### Materials

Materials, data and code are available on the OSF, https://osf.io/5z49n/.

#### Target words

Target words in the Ambiguous condition (e.g., “organ”) were noun-noun homophones selected from recent word association norms from British English speakers (Gilbert & Rodd, 2022) where the subordinate meaning was depictable in a single image (*N* = 66). Unambiguous nouns were selected pairwise for the Unambiguous (item-matched) condition to be sufficiently similar to their matched Ambiguous target that they could be depicted by the same picture (e.g., “piano”; Table 2). Unambiguous nouns were also selected as target words for the Unambiguous (set-matched) condition (e.g., “leather”). Target words in the Unambiguous (set-matched) condition were chosen from a pool of candidates produced by the LexOPS shiny app (https://jackt.shinyapps.io/lexops/, Taylor et al., 2020), with parameters set to match the ambiguous target on frequency (+/– 1000 words per million; SUBTLEX-UK database; van Heuven et al., 2014), number of syllables (calculated using eSpeak speech synthesiser; http://espeak.sourceforge.net/) and age of acquisition (+/– 0.5 years; Kuperman et al., 2012). From this pool, we manually selected singular nouns that could be used at the end of a story without repeating themes across narratives. Although the sets of ambiguous and unambiguous stimuli were well matched overall on frequency (see Table 2), it was necessary to include some pairs where the items within a pair differed on frequency by up to 334 words per million (e.g., “play” has frequency of 489.02 words per million; “phone” has frequency of 155.13).

**Table 2:** **Descriptive Statistics.** Frequency is given in frequency per million words, based on SUBTLEX-UK (van Heuven et al., 2014). The table contains frequency means, with standard deviations in brackets. Age of acquisition and Familiarity ratings were taken from Scott et al., 2019. Number of syllables was calculated for British pronunciations, by eSpeak speech synthesiser (http://espeak.sourceforge.net/). Narrative and Picture information was newly collected (see below).


	AMBIGUOUS (N = 66)	UNAMBIGUOUS (ITEM-MATCHED) (N = 66)	UNAMBIGUOUS (SET-MATCHED) (N = 66)

Target word characteristics			

Frequency	43.72 (74.16)	34.32 (58.41)	42.39 (97.59)

Age of acquisition	3.23 (0.87)	3.48 (1.18)	3.00 (0.85)

Familiarity	5.65 (0.79)	5.78 (0.68)	5.70 (0.75)

Number of syllables	0.97 (0.86)	1.71 (1.15)	1.17 (0.82)

Narrative characteristics			

Number of words	25.04 (3.69)	25.04 (3.69)	24.15 (3.79)

Narrative naturalness rating	5.27 (1.66)	5.27 (1.66)	5.27 (1.67)

Key word fit: LSA score	0.07 (0.11)	0.07 (0.09)	0.08 (0.10)

Target picture characteristics			

Picture representativeness	4.93 (1.78)	5.16 (1.78)	5.74 (1.48)


**Narrative contexts**

A three-sentence story was created for each ambiguous target word (see Table 1). Sentence 1 provided a situational context in which the subordinate meaning of the ambiguous noun was more plausible than its dominant meaning. Sentence 3 ended with the ambiguous target word. Sentences 2 and 3 were compatible with both meanings of the ambiguous word, and thereby contributed minimal disambiguating information.

Narratives in the Unambiguous (item-matched) condition were identical to the Ambiguous condition, except that the final target word was replaced with an appropriate unambiguous target, i.e., “piano” replaced “organ” (Table 1). Unambiguous (set-matched) narratives followed the same general structure. The stories were comparable across conditions in terms of the types of topics discussed, approximate sentence length and the level of vocabulary used.

Individual sentences were recorded by a female speaker of Southern British English at a sampling frequency of 44.1 kHz. Sound files were processed in Praat (v 6.1.16, Boersma & Weenink, 2020). All sentence sound files began with approximately 30 ms of silence before speech onset, and were cut at speech offset. All sound files were down-sampled to 22,050 kHz, the intensity was scaled to 70 dB, and they were band-pass filtered from 60–20,000 Hz with a smoothing factor of 10. Files were converted into stereo .mp3 files using Audacity (v2.3.3).

Participants rated the naturalness of the audio narratives in a pre-test set up using Gorilla Experiment Builder (www.gorilla.sc/about; Cauldron Inc.; Anwyl-Irvine et al., 2019). They were recruited via Prolific Academic (www.prolific.co) from the same pool as the main experiment. Participants were not permitted to take part in multiple pre-tests (see Supplementary Materials for details). They rated the narratives on a 1–7 Likert scale on the basis of how “natural each story [felt to them], based on whether it flows well and makes sense”. The final target words were removed from the narratives so that ratings would not be influenced by whether disambiguation was required. The items for the Ambiguous and Unambiguous (item-matched) conditions were therefore identical. We analysed data from 60 participants (30 participants per item). Naturalness ratings were relatively high and there were no statistically significant differences between the Ambiguous/Unambiguous (item-matched) narratives and those in the Unambiguous (set-matched) condition (Table 2; *t*(130) = 0.02, *p* = 0.99).

In addition, we used Latent Semantic Analysis (LSA; Landauer & Dumais, 1997) to establish how well the target word fitted with the preceding narrative (see Supplementary Material for details). The output from this procedure is a score between –1 (very low semantic similarity) and 1 (very high semantic similarity). We observed low scores (ranging from –0.11 to a maximum of 0.5), reflecting the relatively low predictability of the target words in these contexts. There was no significant difference in target word fit between the three conditions (Table 2; *F*(2,191) = 0.153, *p* = 0.86).

#### Picture probes

Pictures were taken from the Noun Project icon database (https://thenounproject.com/). The 66 ambiguous words were paired with a single black-and-white picture that depicted both its subordinate meaning (e.g., the musical meaning of “organ”) and its counterpart in the Unambiguous (item-matched) condition (e.g., “piano”). A non-overlapping set of pictures were chosen for the 66 target words in the Unambiguous (set-matched) condition (e.g., “leather”). Each target picture was paired with an unrelated picture that served as its distractor in the two-alternative forced choice task. Distractors were selected from a list of items generated by a random noun generator (https://randomwordgenerator.com/) on the basis of being relatively unambiguous, easily imageable and unrelated to their narrative. Each target picture was paired with a distracter with similar visual complexity. To ensure participants’ attention throughout the narratives and to avoid response strategies focused on narrative-final words only participants had to respond to a second two-alternative forced-choice prompt earlier in each experimental narrative. At the offset of Sentence 1, two picture probes appeared, with one picture clearly related to the sentence and an unrelated distracter picture. For example, the sentence “The shop had some complicated items that needed repair” was followed by a picture depicting “repair” and a distracter picture (see also Figure 1). These Sentence 1 picture probes also allowed us to make comparisons between conditions at a point in the narrative where we would not expect statistical differences (i.e., because the sentence and pictures were identical in the Ambiguous and Unambiguous (item-matched) condition, or because we have no theoretical reason to justify processing differences between the Ambiguous and Unambiguous (set-matched), or between the two control conditions).

A pre-test was used to establish the extent to which pictures represented the intended words. Participants saw word-picture pairs (e.g., a picture of a piano/organ alongside either the word *piano* or the word *organ*) and were asked to rate how well the picture represented the word’s meaning on a scale from 1 (“represents the word’s meaning not at all”) to 7 (“represents the word’s meaning perfectly”, see Supplementary Material). Each target word was presented with a short definition of its contextually appropriate meaning to make sure that ambiguous target words were rated in relation to the intended meaning. We analysed data from 90 participants (30 participants per word-picture pair). A one-way ANOVA revealed a significant difference in mean ratings between conditions, *F*(2, 195) = 15.05, *p* < .001, η^2^ = 0.13. Post-hoc tests showed that the picture-word pairs in the Unambiguous (set-matched) condition (M = 5.74, SD = 1.48) were rated more highly than items in both the Ambiguous (M = 4.93, SD = 1.78) and Unambiguous (item-matched) condition (M = 5.16, SD = 1.78; see Table 2; both *p* < .001). This likely indicates that the additional constraints on selecting pictures that worked for both the Ambiguous and Unambiguous (item-matched) conditions had resulted in the selection of pictures that were less optimal than those chosen in the less (set-matched) constrained Unambiguous condition. Note that there was no significant difference between picture-word representative ratings across the Ambiguous and Unambiguous (item-matched) conditions.

### Filler narratives

We included filler narratives to make the timing of the picture probes within each narrative less predictable and less open to strategic influences. We developed 13 filler narratives with similar characteristics to those in the three conditions. Picture probe pairs were selected for each narrative. These were presented at the onset of Sentence 1 in nine filler narratives and at the offset of Sentence 2 in the other four filler narratives.

### Design

Participants completed a 2-alternative-forced-choice picture selection task, within a repeated-measures two-version design. Accuracy and response times of button presses in response to picture probes were our dependent measures. Each participant encountered half the items (33 narratives) from each of the Ambiguous, Unambiguous (item-matched) and Unambiguous (set-matched) conditions. Two stimulus lists (or versions) were created such that the Ambiguous and Unambiguous (item-matched) variants of the same narrative were counterbalanced across participants, and no participant encountered the same narrative twice. Each list also included the 33 Unambiguous (set-matched) narratives that were pair-wise matched to Ambiguous narratives in that list. All participants encountered the same 13 filler stories.

### Procedure

The experiment was set up using the Gorilla Experiment Builder (www.gorilla.sc/about; Cauldron Inc.; Anwyl-Irvine et al., 2019). Participants completed it online in their own time, using their own computer or laptop with a keyboard and headphones. Participants first completed an audio technology check. They were asked to confirm that their browser’s auto-play function for audio files was enabled, or, if this was not the case, they were shown how to enable this function (based on a Gorilla task developed by Milne et al., 2021). They then completed a six-trial Huggins Pitch task (also developed by Milne et al., 2021) in which they heard three bursts of white noise and were asked to identify a hidden ringing tone that was only audible when wearing headphones. In the initial phase of data collection, participants could only continue on to the main task if they had passed all six Huggins Pitch trials. We experienced a high level of data loss due to this restriction (n = 36 out of 88 participants who started the experiment), and therefore decided to change the restriction such that participants could continue on to the main task if they had passed four out of six trials.

Participants then heard an example sound file from the same recording session as the files used in the main task, and adjusted their volume levels. They were asked to turn off background noise on their device (e.g., notifications, music) and to maximise their browser window. They completed a brief demographic questionnaire on their language background, age, and sex.

The main task was a picture selection task. Participants were told that they would hear a succession of short stories and that within each, they would sometimes see a pair of pictures on the screen, and that they should then “quickly select the picture that fits best with what [they had] just heard in the story” (“c” vs “m” keys for the picture presented on the left and right, respectively). They were told that they should do this as quickly and accurately as possible (Figure 1).

Each trial started with a fixation cross, presented in the centre of the screen for 1500 ms, with 100 ms of blank screen both before and after the cross appeared. Each spoken sentence was presented individually, and played automatically. Following Sentence 1 in each trial, a 100 ms buffer blank screen appeared. To ensure participants’ attention and serving as a “sanity check” for our analysis of condition differences, two pictures then appeared (coinciding with the auditory presentation of Sentence 2). One of these pictures was related to the final word in Sentence 1, (e.g. “repair”) while the other was an unrelated distracter. Button press response times to these Sentence 1 picture probes were measured from picture onset, i.e. 100 ms after sentence offset. Picture probes disappeared from the screen automatically after 3000 ms, or at the offset of Sentence 2 (whichever occurred first). Sentence 2 was followed by another 100 ms buffer blank screen, and then by Sentence 3. The critical picture probes appeared on the screen at the offset Sentence 3, i.e. after the final target word. The target picture was related to the intended (subordinate) meaning of the ambiguous word (or the meaning of the unambiguous word in the control conditions), while the distracter was unrelated. Button press response times to these picture probes were measured from picture onset, i.e. at the offset of the target word. Pictures disappeared and trials timed out after 3000 ms.

Filler narratives followed the same basic pattern, with fixation cross and buffer screens between sentences. However, picture probes were presented either at the onset of Sentence 1, or 100 ms after the offset of Sentence 2. Button press data for these probes were collected but not analysed.

Before the main task, participants completed four practice trials with feedback. After the practice trials, the main task always began with two filler narratives, so that only data from the third trial onwards were analysed. Otherwise, stimulus presentation order was randomized for each participant. The position of target and distracter pictures on the left vs the right of the screen was randomly assigned for each item (50% of targets on the left overall) but kept the same for all participants.

## Results

### Group-level analyses

Our aims in this section were twofold. Firstly, we test our hypothesis that responses to the critical picture probes following Sentence 3 would be less accurate and slower in the Ambiguous condition compared to the Unambiguous (item-matched) condition, replicating the “subordinate bias effect” using a typical psycholinguistic experimental design. The second aim was to test the appropriateness of the Unambiguous (set-matched) condition (compared to the Unambiguous (item-matched) condition) for future studies of individual differences in disambiguation ability.

#### Group-level analyses: analytic approach

We ran three sets of group-level analyses for picture selection accuracy and response times (for correct selections): 1) Ambiguous vs. Unambiguous (item-matched), 2) Ambiguous vs. Unambiguous (set-matched), 3) Unambiguous (item-matched) vs. Unambiguous (set-matched; see “Group-level” analysis in Table 3). Because the three conditions differed in the representativeness of picture-word ratings of the critical probes, we included centred mean picture rating for each item as a continuous covariate in our models.

**Table 3:** Summary of analysis aims and statistical models. For simplicity, covariates are not included in this summary. Amb = Ambiguous, Unamb = Unambiguous.


ANALYSIS	CONDITION COMPARISON	AIM	MAXIMAL MODEL	COMPARISON MODEL

**Group-level**	Amb vs Unambi (item-matched)	Replicate ambiguity effect using Unambiguous (item-matched)	1 + Condition + List + Condition:List + (1 + Condition|subjects) + (1 + Condition|items)	1 + List + Condition:List + (1 + Condition|subjects) + (1 + Condition|items)

	Amb vs Unamb (set-matched)	Replicate ambiguity effect using Unambiguous (set-matched)	1 + Condition + (1 + Condition|subjects) + (1|items)	1 + (1 + Condition|subjects) + (1|items)

	Unamb (item-matched) vs Unamb (set-matched)	Ensure control conditions are comparable	1 + Condition + (1 + Condition|subjects) + (1|items)	1 + (1 + Condition|subjects) + (1|items)

**Individual differences**	Amb vs Unamb (set-matched)	Assess individual differences in task performance	1 + Condition + (1|subjects) + (1|items)	1 + Condition + (1|items)

	Amb vs Unamb (set-matched)	Assess individual differences in ambiguity effect	1 + Condition + (1 + Condition|subjects) + (1|items)	1 + Condition + (1|subjects) + (1|items)


Filler narrative trials and practice trials were removed prior to data analysis. We analysed accuracy and response times on first attempts at a button press only, and removed trials with response times below 250 ms (2.90% of the first-attempt button presses). We retained trials that were timed out for accuracy analyses, counting these trials as errors. On critical picture probes (i.e. probes that appeared after Sentence 3), time-outs were defined as trials in which a response did not occur within a 3000 ms time window from appearance of the pictures (0.23%). For response time analyses, we removed all incorrect trials (including time-outs). We checked the assumption of normality of residuals by visual inspection of predicted-residual plots and histograms of raw data, inverse-transformed and log-transformed response time data. As the log-transformation provided the most appropriate distribution of residuals all response times were log-transformed prior to analysis (Baayen, 2008; Brysbaert & Stevens, 2018). Data were analysed using mixed effects models in R (version 3.6.2) within RStudio (version 1.4.1106; RStudio Team, 2015), using the glmer() and lmer() functions from the lm4 package (version 1.1-26; Bates et al., 2015). We fitted mixed effects models with maximal random effects structures, following Barr and colleagues (2013).

1. Ambiguous vs Unambiguous (item-matched). Due to counterbalancing, the paired narratives in the Ambiguous and Unambiguous (item-matched) condition were encountered by different participants across the two stimulus lists. For comparisons between these two conditions, we therefore fitted mixed effects models with a fixed effect for Condition (deviation-coded as Ambiguous -0.5/Unambiguous (item-matched) 0.5), a fixed effect for the between-subjects factor List (deviation-coded List A 0.5/List B –0.5) and a fixed Condition × List interaction effect. The random effects structure was kept maximal where possible, with a random intercept and random slope for Condition by subjects, and a random intercept and random slope for Condition by items.
2. Ambiguous vs Unambiguous (set-matched). The Ambiguous condition and Unambiguous (set-matched) condition differed in narrative context, probe words and the pictures that were presented. Each participant encountered Ambiguous and Unambiguous (set-matched) trials, but the items did not constitute pairs. For comparisons between the Ambiguous and Unambiguous (set-matched) condition, we therefore fitted mixed effects models with a fixed effect for Condition (deviation-coded as Ambiguous –0.5/Unambiguous (set-matched) 0.5). The random effects structure was kept maximal, with random intercept and random slope for Condition by subjects, and a random intercept by items.
3. Unambiguous (item-matched) vs Unambiguous (set-matched). The Unambiguous (item-matched) condition and Unambiguous (set-matched) condition differed in narrative context, probe words and the pictures that were presented. For comparisons between the two control conditions, we therefore fitted mixed effects models with a fixed effect for Condition (deviation-coded as Unambiguous (item-matched) –0.5/Unambiguous (set-matched) 0.5). The random effects structure was kept maximal, with random intercept and random slope for Condition by subjects, and a random intercept by items.

In the case of non-convergence or singular fit, the random effect structure of a model was reduced following these steps: 1) removing the correlations between random slopes and random intercepts, 2) removing random intercepts. If these steps did not solve the convergence issues, we started from the maximal random effects structure again, and removed the random slope that accounted for the least amount of variance. If this step also did not solve the issue, we followed the above steps 1 and 2 again, having removed one of the random slopes. We followed these steps iteratively until the model converged, and then used the maximal model that would converge for all model comparisons via likelihood ratio tests.

#### Group-level analyses: Results

**Figure 2:**
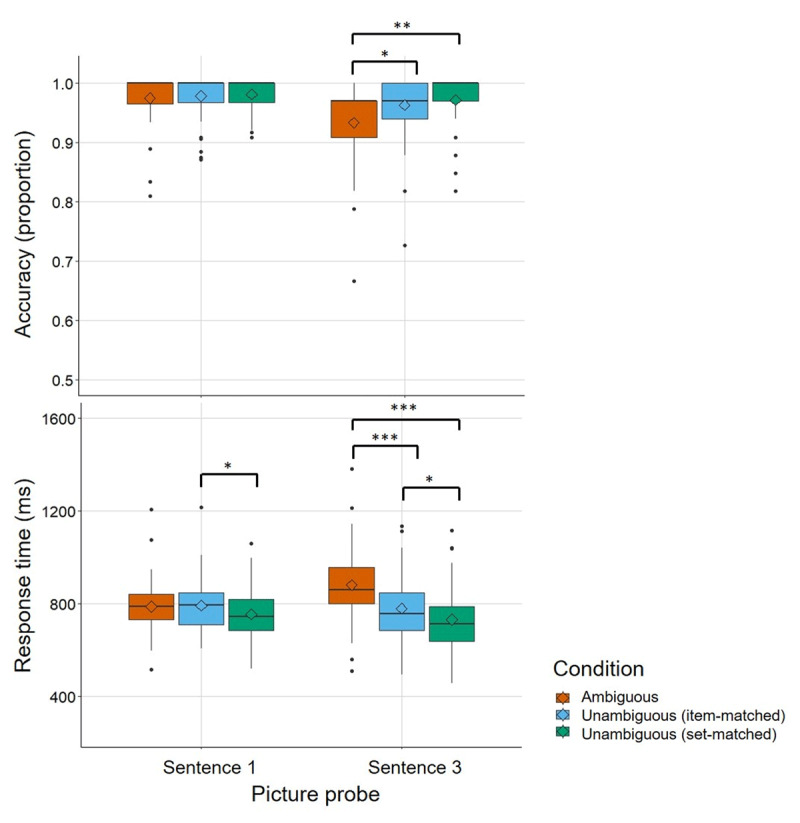
Mean accuracy (proportion) and response time on correct trials (ms) in each of the three conditions on Sentence 1 and Sentence 3 probes for each participant. Boxplots show median and quartiles, diamond shows mean across the sample. * *p* < .05; ** *p* < .01; *** *p* < .001’

Before reporting results from the critical picture probes, we first report analyses of accuracy and response times of the picture probes that followed Sentence 1. This allowed us to confirm that participants paid adequate attention to the stories and that there were no salient differences between conditions at this point in the narratives. We followed the same data cleaning and transformation procedures as for the other picture probes described above, but time-outs were defined as trials in which a response did not occur within the duration of the Sentence 2 audio file. There were no such time-outs within the dataset. Descriptive data are shown on the left panels in Figure 2.

We first compared the Ambiguous and Unambiguous (item-matched) conditions, where narrative and picture probe were identical for matched pairs. Main effects of Condition or List, and the Condition × List interaction were non-significant for accuracy (all *p*s > .2) and response times (all *p*s > .3).

Next, we compared the Ambiguous and Unambiguous (set-matched) conditions, where narratives were non-identical but unambiguous at this point. The main effect of Condition was non-significant for both accuracy (*p* = .601) and response times (*p* = .057).

Finally, the comparison across the two control conditions (Unambiguous (item-matched) vs. Unambiguous (set-matched)) revealed no main effect of Condition on accuracy (*p* = .331). For response times, however, the main effect of Condition was significant, b = 0.02, SE = 0.01, t = 2.53, χ^2^(1) = 6.38, *p* = .012, with significantly longer response times in the Unambiguous (item-matched) (M = 784.18, SD = 223.5) compared to the Unambiguous (set-matched) condition (M = 747.99, SD = 204.01).

Performance on the critical Sentence 3 picture probes is plotted in the right panels of Figure 2. First, we will discuss the comparison between the Ambiguous and Unambiguous (item-matched) condition, which had the aim of replicating the classic “subordinate-bias effect”. As predicted, there was a significant main effect of Condition on accuracy, b = 1.03, SE = 0.46, z = 2.24, χ^2^(1) = 5.42, *p* = .02, with significantly lower accuracy in the Ambiguous (M = 0.93, SD = 0.25) compared to the Unambiguous (item-matched) condition (M = 0.96, SD = 0.19). The main effect of List was also significant, b = –1.18, SE = 0.45, z = –2.65, χ^2^(1) = 7.28, *p* = .007, with List A associated with significantly lower accuracy (M = 0.94, SD = 0.25) than List B (M = 0.96, SD = 0.19). The Condition × List interaction was significant, b = –1.39, SE = 0.70, z = –1.97, χ^2^(1) = 4.07, *p* = .044. The main effect of the picture representativeness rating covariate on accuracy was also significant, with those items that had received higher picture ratings associated with relatively greater accuracy in the disambiguation task, b = 0.39, SE = 0.16, z = 2.47, χ^2^(1) = 6.18, *p* = .013. None of the interactions of picture rating with the other fixed effects were significant (all *p*s > .1). As predicted, the main effect of Condition on response times was also significant, b = –0.04, SE = 0.01, t = –7.41, χ^2^(1) = 41.68, *p* < .001, with significantly longer response times in the Ambiguous condition (M = 879.47, SD = 311.04) compared to the Unambiguous (item-matched) condition (M = 781.73, SD = 254.99). The main effect of List and the Condition × List interaction were non-significant (*p*s > .2). The main effect of the picture rating covariate was significant, b = –0.03, SE = 0.004, t = –6.59, χ^2^(1) = 38.63, *p* < .001, as was the Condition × Picture rating interaction, b = 0.02, SE = 0.01, t = 2.8, χ^2^(1) = 7.54, *p* = .006. Post-hoc comparisons with Tukey-adjusted p-values using the emmeans() package (v. 1.6.2-1, Lenth, 2021) showed that the slope for picture rating was significantly steeper in the Ambiguous condition than the Unambiguous (item-matched) condition. Items with higher picture representativeness ratings were associated with relatively faster response times in the disambiguation task (b = –0.02, SE = 0.01, z-ratio = –2.89, *p* = .004; averaged over lists).

The comparison between the Ambiguous condition and the Unambiguous (set-matched) condition had the aim of establishing this less well-matched control condition as a viable alternative to a closely-matched control for use in individual differences designs. As predicted, the main effect of Condition on accuracy was significant, χ^2^(1) = 7.39, *p* = .007, with lower accuracy in the Ambiguous condition (M = 0.93, SD = 0.25) compared to the Unambiguous (set-matched) condition (M = 0.97, SD = 0.17). The main effect of picture rating (*p* = .06) and the Condition × Picture rating interaction (*p* = .805) were non-significant. There was also a significant main effect of Condition on response times, b = –0.06, SE = 0.01, t = –5.55, χ^2^(1) = 28.38, *p* < .001, with significantly longer response times in the Ambiguous condition (M = 879.47, SD = 311.04) compared to the Unambiguous (set-matched) condition (M = 731.82, SD = 253.91). The main effect of picture rating on response times was also significant, b = –0.03, SE = 0.01, t = –4.39, χ^2^(1) = 18.38, *p* < .001, with those items that had received higher picture representativeness ratings in our pilot associated with relatively faster response times in the disambiguation task. The Condition × Picture rating interaction was non-significant (*p* = .211).

Finally, we compared the two control conditions. We expected there to be no significant difference in accuracy or response times between the two control conditions, thereby supporting the conclusion that the differences between the Ambiguous and Unambiguous (set-matched) condition were likely driven by the difference in terms of disambiguation demands, rather than simply driven by differences in the lexical items, sentence structure, or pictures used. As anticipated, there was no main effect of Condition on accuracy; nor was there a main effect of picture rating or interaction (all *p*s > .09). In contrast, the main effect of Condition on response times was significant, b = 0.02, SE = 0.01, t = 2.31, χ^2^(1) = 5.38, *p* = .02, with significantly longer response times in the Unambiguous (item-matched) (M = 781.73, SD = 254.99) compared to the Unambiguous (set-matched) condition (M = 731.82, SD = 253.91). The main effect of picture rating was also significant, b = –0.02, SE = 0.01, t = –3.81, χ^2^(1) = 14.16, *p* < .001, with those items that had received higher picture representativeness ratings being associated with relatively faster response times in the disambiguation task. The Condition × Picture rating interaction, however, was non-significant (*p* = .793).

#### Interim summary

Our analyses replicated the classic “subordinate-bias effect” with lower accuracy and longer response times in narratives containing an ambiguous word compared to otherwise identical narratives without the ambiguous word (Unambiguous (item-matched) condition). The same pattern was seen for the comparison between ambiguous narratives and those in the less well-matched unambiguous condition. This allows us to be optimistic about the appropriateness of this Unambiguous (set-matched) condition in individual differences designs. Reassuringly, there were no differences across ambiguous vs unambiguous conditions before Sentence 3, indicating that the source of condition differences was related to the ambiguity manipulation itself. Adding complexity, however, a comparison between the two control conditions had mixed results. At the level of picture selection accuracy, the two control conditions behaved similarly. However, response times on both Sentence 1 and Sentence 3 picture probes differed across the two control conditions. We will discuss the implications of these results for the validity of our task in the Discussion.

#### Assessing individual differences

Our aim in this section was to determine whether there were systematic differences between the performance of participants both in terms of (i) their overall task performance (i.e. speed and accuracy of their responses, regardless of condition) and (ii) the extent to which their performance is impacted by condition (i.e. the effect of ambiguity). We consider each of these questions before turning to consider measurement reliability.

##### Individual differences: Analytic approach

We investigated the existence of individual differences in the effect of ambiguity in a comparison between the Ambiguous and Unambiguous (set-matched) condition, following a procedure using mixed effects models (Kliegl et al., 2011; Staub, 2021). In these models, random intercepts by-subjects reveal whether individual participants are consistently slower or more error-prone than others. In addition, we can account for between-subject variation in the effect of a variable-of-interest (e.g., Ambiguity) by including a random slope. We can check whether there are systematic differences in the extent to which individuals are influenced by this variable by comparing a model that includes a random slope (for, e.g., Ambiguity) to a model that does not (i.e., a random-intercepts-only model). A significant improvement in model fit with the inclusion of the random slope would be taken as evidence for the existence of systematic individual differences. We therefore fitted three models with identical fixed-effects but varying random-effects structure to accuracy and log-transformed response time data for the picture selection responses in critical Sentence 3 picture probes: Model 0 without any by-subjects random effects, Model 1 with a random by-subjects intercept, and Model 2 with a random by-subjects intercept and a random by-subjects slope for Ambiguity, allowing intercept and slope to covary.

##### Individual differences: Results

To test for systematic individual differences in overall task performance (i.e. speed and accuracy of responses, regardless of condition), we compared Model 1 to Model 0. For accuracy, Model 1 (AIC: 1137.2, BIC: 1173.8) showed significantly improved fit compared to Model 0 (AIC: 1207.5, BIC: 1238.0), χ^2^(1) = 72.24, *p* < .001. Similarly, for response times, Model 1 (AIC: –5079.1, BIC: –5036.8) showed significantly improved fit compared to Model 0 (AIC: –3994.3, BIC: –3958.0), χ^2^(1) = 1086.9, *p* < .001 These results provide evidence for systematic individual differences in overall accuracy and response time, independent of condition, on the picture selection task.

To test for systematic individual differences in the extent to which participants’ performance was impacted by condition (i.e. the effect of ambiguity), we compared Model 2 to Model 1. For accuracy, we found a significant improvement in fit for Model 2 (AIC: 1132.5, BIC: 1181.3) over Model 1 (AIC: –5079.1, BIC: –5036.8), χ^2^(2) = 8.76, *p* = .013. This result indicates that there are individual differences in the effect of condition (i.e. ambiguity) on picture selection accuracy. For response times, we did not find a statistically significant improvement in fit for Model 2 over Model 1, χ^2^(2) = 4.20, *p* = .122.

**Figure 3:**
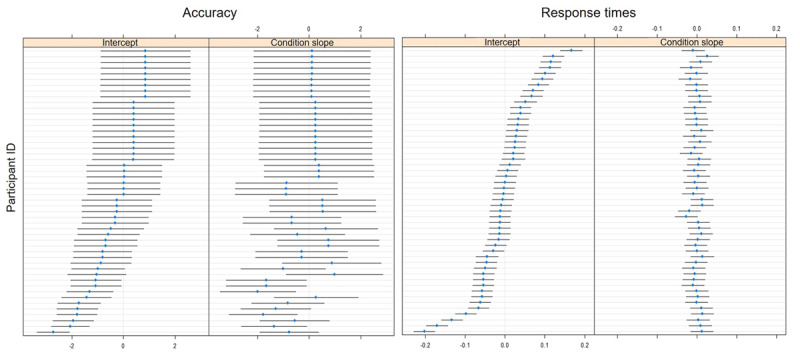
Estimates for individual participants, for the intercept and the condition difference in accuracy and response time of picture selection. We used the dotplot() function from the lattice package, and the ranef() function from lme4, to plot conditional modes from the maximal model (i.e. “predictions” for means of individual participants, based on the parameter estimates of our model). Each row represents the conditional mode (and standard error) for one participant, in terms of its deviation from the population mean (centred at 0). Individual participants are rank-ordered according to the conditional mode of their intercept, from highest estimate (i.e., positive deviation from the mean) to lowest (i.e., negative deviation from the mean).

Figure 3 shows conditional modes from Model 2 (the maximal model) for each participant. These conditional modes are “predictions” of the means of individual participants, based on parameter estimates of our model. We plotted conditional modes for the intercept (reflecting overall differences in task performance between participants) and for the random slope for condition (reflecting individual differences in the effect of ambiguity on the dependent variable). As can be seen on left-hand ‘Accuracy’ and ‘Response times’ panels of Figure 3, there is a degree of individual variability in mean accuracy and in mean response time on the task, as intercept values for individuals deviate from the 0-line (i.e. the population mean). In the right-hand panel ‘Accuracy’ of Figure 3, there is also an indication of individual variability in the size of ambiguity effects on accuracy, with individual conditional modes deviating from the 0-line (i.e. the population mean) of the Condition effect. However, there is no clear evidence for individual variability in the size of ambiguity effects on response times since individual conditional modes do not deviate far from the 0 line of the Condition effect.

### Task reliability

The model comparisons in the previous section provided evidence for the existence of systematic individual differences in the influence of ambiguity on picture selection accuracy. What is still unclear is whether an individual difference measure derived from the task could be successful in rank-ordering participants consistently. We therefore asked whether the task has shown adequate measurement reliability in this sample. We calculated reliability estimates by employing a permutation approach to split-half reliability, following Parsons et al., (2019). We report reliability estimates for each condition (Ambiguous, Unambiguous (item-matched), Unambiguous (set-matched)) in Table 4. Using the splithalf package (v.0.7.1, Parsons, 2021) in R, we created 5000 random splits of the trial-level data, and for each split calculated correlations between averages for each half of the data for each participant. We report here the Spearman-Brown corrected mean correlation coefficient and 95% confidence interval from this procedure. As can be seen in Table 4, reliability estimates for the response time measure are excellent, while estimates for accuracy are lower with much wider confidence intervals.

**Table 4:** **Reliability estimates for each dependent variable and condition.** Estimates are Spearman-Brown corrected mean correlation coefficients (and 95% confidence intervals) based on 5000 random splits of the data (Parsons, 2021).


	RELIABILITY ESTIMATE	

	ACCURACY	RESPONSE TIME (LOG-TRANSFORMED)

**Averages**		

Ambiguous	0.67, 95% CI [0.48, 0.81]	0.92, 95% CI [0.88, 0.95]

Unambiguous (item-matched)	0.69, 95% CI [0.47, 0.83]	0.93, 95% CI [0.90, 0.95]

Unambiguous (set-matched)	0.67, 95% CI [0.40, 0.85]	0.93, 95% CI [0.90, 0.96]

**Difference scores**		

Ambiguous – Unambiguous (set-matched)	0.43, 95% CI [0.07, 0.67]	0.14, 95% CI [–0.22, 0.47]


In individual differences studies using subtractive designs, researchers often calculate a “difference score” between conditions to isolate the process of interest and form a single score for each participant. For example, we may want to average each participants’ response times in the Ambiguous condition, and subtract from that their average response time in the Unambiguous condition, to get at their “ambiguity effect”. We end our investigation of reliability therefore by considering the reliability estimate for difference scores between the Ambiguous and Unambiguous (set-matched) condition (i.e. capturing variation in disambiguation performance within the same participant across different narratives), using the same permutation-based split-half procedure. As can be seen in Table 4, reliability estimates are relatively poor for difference scores based on accuracy, and even worse for response time difference scores. These results strongly discourage the use of such difference scores in future investigations of individual differences with our disambiguation task. Note, however, that results from the group-level analysis of the present data do not hinge on the calculation of difference scores.

## Discussion

Adapting group-level experimental designs and applying them to the study of individual differences is far from straightforward (Hedge et al., 2018; Rouder et al., 2019; Rouder & Haaf, 2019). There are foundational differences in design logic between these two research domains, and they present challenges with respect to both validity and reliability (the “Yin and Yang of any undergraduate textbook on research methods”; Holmboe, 2022).

In the current study we adapt a group level task to develop a task suitable for measuring individual differences in participants’ ability to use contextual cues to guide word-meaning access. This work highlights the specific complexities that arise when adapting experimental paradigms (i) that use a subtraction design that compares performance on a condition-of-interest with a control condition, and (ii) for which multi-version designs are typically used to ensure appropriate stimulus control between conditions. In this discussion we reflect on various design decisions and compromises, and use our results to consider the impact of our approach and possible lessons for future work.

### Is our Task Valid?

At the heart of our experiment is the comparison between performance in the Ambiguous and Unambiguous conditions. To be informative at the level of individual differences, these conditions needed to be sufficiently dissimilar so that *all* participants could respond to *all* items. By definition, a single-version experimental design precludes the very close item-to-item matching across conditions that is typical in traditional multi-version experiments on lexical ambiguity (e.g., placing target words within identical sentence frames). In the present study, the Ambiguous vs Unambiguous (set-matched) conditions therefore included different sentence frames with non-overlapping meanings, and were paired with entirely different picture probes. We matched as best we could without repeating any linguistic content that would likely lead to between-item priming effects, but acknowledge that the threat to measurement validity caused by this mismatch between experimental and control condition cannot be eliminated entirely, but only contained as best we can. Our aim in this experiment was to assess the utility of this “unmatched” Unambiguous condition to then have confidence in using it in future larger-scale studies of individual differences.

Our primary approach to establishing the utility of this comparison between our conditions of interest was to include an additional control condition, namely one that was more closely matched to the Ambiguous condition. To do this, we drew on the standard condition used in group-level studies of ambiguity resolution where, other than the target word (“organ” vs “piano” in our example), the context is kept identical across conditions. Performance in the Ambiguous condition could then be compared directly with both this Unambiguous (item-matched) condition and the Unambiguous (set-matched) condition. In an ideal world, the two control conditions would behave in an identical manner in terms of both their group-level and participant-level characteristics – any observed differences between them would reflect imperfect stimulus matching between our conditions. We collected extensive pilot and norming data to explore item-level characteristics and to minimise any differences between conditions that might influence performance.

With respect to the potential utility of the Unambiguous (set-matched) condition as a control, the results are mixed. The Unambiguous (set-matched) and Unambiguous (item-matched) conditions are similar in that both revealed an appropriate ‘ambiguity disadvantage’ (or “subordinate bias effect”; Pacht & Rayner, 1993; Rayner et al., 1994) relative to the Ambiguous Condition. This effect was seen in both the accuracy and latency data (Figure 2). Contrary to our expectations, however, the Unambiguous (set-matched) condition was associated with a numerically larger ambiguity effect than the Unambiguous (item-matched) condition. Of particular concern, a direct statistical comparison between these two supposedly equivalent control conditions revealed that responses were faster in the Unambiguous (set-matched) condition. This relatively small, but statistically significant, condition difference is difficult to explain. We worked through several iterations of stimulus generation, pre-testing and selection, yet we were still left with undesired differences by condition that cannot be attributed to any obvious difference between the two. Conditions were well-matched in terms of rated naturalness, and for the fit between the narrative context and the target word – at least as measured by LSA. It is worth noting the potential of sophisticated Large Language Models (e.g., the GPT family of models) to shape approaches to stimulus matching in language research, particularly when researchers face multiple and complex contextual constraints as they develop stimuli. Our stimuli did differ with respect to how well the target pictures represented the meaning of the target word, but the overall difference in performance between the two control conditions persisted, even when picture ratings were included as a covariate in the analysis.

Despite our best efforts, there must be differences between the two conditions that were systematic enough to produce significant differences in performance. Of course, there are numerous additional variables that we did not consider. In an ideal world, variation in additional variables would distribute randomly across conditions such that systematic differences in performance across conditions would be unlikely. Unfortunately, however, as anyone who has ever created psycholinguistic stimuli is all too aware, this assumption is rarely safe. Language stimuli vary on numerous dimensions that often correlate such that when one dimension is manipulated, we inadvertently induce variation in another correlated dimension and it might be this complex interplay between stimulus characteristics that influences behaviour. This issue has been widely discussed: over 40 years ago, Anne Cutler (1981) described the history of psycholinguistics as a chronicle of “the continual discovery of new confounds”. Indeed, she used lexical ambiguity as a case study to illustrate this exact point, describing how some of the early reports of ambiguity effects on word processing disappeared when the stimuli were appropriately matched on factors such as word length. Ambiguous words tend to be shorter than unambiguous words meaning that in the absence of explicit matching, length may well serve as a confound. Forty years after Cutler’s important paper, the endeavour to disentangle the effects of highly correlated linguistic variables continues (e.g., Mandera et al., 2020; Miguel-Abella et al., 2022; Siew et al., 2021; Tse et al., 2022).

Returning to the difference between our two control conditions, it remains unclear how this small, but statistically significant difference, came about. Although we cannot be certain, we suggest it is a likely – and perhaps even inevitable – consequence of the constraints we faced during stimulus creation. Multiple simultaneous constraints made construction of the Ambiguous and Unambiguous (item-matched) conditions difficult (i.e., finding matched pairs of words to fit into the same narrative context and be depicted by the same picture). By contrast, the Unambiguous (set-matched) condition was relatively unconstrained and it may be that this resulted in items that were in some hard-to-define way just ‘easier’ than those in the other control condition. We anticipated this scenario and attempted to safeguard as best we could (e.g., by pretesting for naturalness), yet some between-condition differences were clearly present.

While unfortunate, this difference in behaviour between the two control conditions serves to illustrate the importance of our study having included both control conditions. Had we taken the approach of simply presenting results from a comparison between the Ambiguous condition and a within-participant control condition (i.e., the Unambiguous (set-matched) condition) that had been matched as closely as possible, the consequence of imperfect matching would have been hidden from view. We included the Unambiguous (item-matched) condition as a form of “sanity check” as we assessed the utility of the Unambiguous (set-matched) condition for future studies of individual differences. We are pleased to have done so: while additional comparisons may not always tell us what we want to hear, it may at least ensure humility in our inferences. It also reminds us to be cautious in how we interpret findings from group-level studies of ambiguity that have used non-matched item sets. In particular, it highlights the need for caution when interpreting the results of neuro-imaging studies where this approach is common due to the expensive and time-consuming nature of data collection (Kadem et al., 2020; Rodd et al., 2005, 2010, 2012; Vitello et al., 2014; Vitello & Rodd, 2015).

Our task is valid in that the key group-level ambiguity effect did emerge, but the above discussion does highlight possible threats to validity. It is also important to be clear that even if we could be highly confident about the appropriateness of the baseline condition, this is only a necessary and not a *sufficient* condition to ensure task validity. Additional work still needs to be done to ensure that participant variation in task performance can confidently be attributed to differences in ambiguity resolution skill. One possibility is to derive, where possible, multiple measures from within the same dataset. For example, the current dataset provides the potential to explore within-condition differences such as the effect of word-meaning dominance (i.e. meaning frequency; Twilley, Dixon, Taylor & Clark, 1994) on performance. Just as narratives containing ambiguity should place more demands on disambiguation processes compared with relatively unambiguous narratives, we would also expect ambiguous narratives whose intended meanings are strongly subordinate (e.g., the “animal enclosure” meaning of “pen”) to show a stronger effect than narratives based on word meanings that are more common. Convergent evidence from within the same dataset may, to some extent, support inferences about the likely factors that are driving any observed individual differences.

Another possibility comes from using multiple measures as indices of the construct-of-interest. In other domains, multiple measures allow psychometricians to capture an underlying trait using a latent variable structural equation model approach in which irrelevant task-specific behaviours that are not relevant to the construct-of-interest can be discounted (e.g. Goh et al., 2021; Dolean et al., 2021; Eid, Lischetzke, & Nussbeck, 2006). It would be optimal to devise multiple tests of disambiguation skill that are likely to load on a common construct-of-interest, but differ in extraneous dimensions such as the details of the task and stimuli.

### Is our Task Reliable?

Our focus so far has been with validity: to what extent can the observed differences in performance across the different conditions be attributed to differences in the targeted cognitive process of disambiguation skill, rather than some other extraneous difference between conditions? Of equal importance is the issue of reliability: to what extent does our task serve as a reliable measurement instrument?

Our findings offer some reassurance on reliability. Reliability estimates were excellent for response times, and near the desired 0.7 mark for accuracy. This might be taken as reassurance that, when adapted for use in a large-scale individual differences study, the combination of task and stimuli developed here will provide a reliable measurement instrument. However, there are several reasons to treat this reliability estimate with caution. First, it is likely that our findings *under estimate* the reliability of the task due to design choices that were made to optimise the ability of our experiment to also address a group-level question. For example, item order was randomised for each participant, and participants were assigned to one of two alternative versions that included different subsets of the stimuli. In addition, any measure of task reliability is necessarily tied to the particular participant sample – there is no guarantee that reliability will generalise to a different participant sample, who may differ in overall levels of performance or sample variance (Parsons et al., 2019). Nonetheless, we strongly advocate that the type of evidence gathered in our study provides an important pre-requisite. Before embarking on a resource-intensive large-scale individual differences study in which performance on a given task is to be compared to other measures to address specific theoretical questions, it is important to establish reliability. Although discussion of individual differences in language skills is prevalent in the literature (e.g., Federmeier & Kutas, 2005; Gernsbacher, 1993; Khanna & Boland, 2010; Lee & Federmeier, 2011; Nation & Snowling, 1998; Norbury, 2005; Snedeker & Trueswell, 2004; Trueswell et al., 1999), few studies that utilize an experimental approach consider reliability, or report the reliability of their measures. As Parsons and colleagues (2019) argue, it is paramount for such research to *always* include an indicator of the reliability of a given task in the current dataset.

Although the internal reliability of a measure is orthogonal to the number of people tested, sample size is an important factor to consider in studies where two (or more) measures are related to each other. We believe that the case study reported here allowed us to adequately address our research questions about the validity of our task and to establish evidence for the existence of individual differences in task performance. However, correlational research questions about relationships between different language skills, or between language and non-linguistic cognitive skills, can be answered appropriately only with internally reliable measures – and enough statistical power to detect such relationships (Spearman, 1910; see also Hedge, Powell, & Sumner, 2018). Deciding on appropriate sample sizes for individual differences studies *a priori* is unfortunately not straight forward. Although there exist some rule-of-thumb recommendations (e.g., N = 150 in Little, 2013; N = 250 in Schonbrodt & Perugini, 2013), a more systematic and customisable approach may lie in simulations based on pilot data of the tasks under investigation (e.g., using the simr package in R, Green & MacLeod, 2016, though see e.g. Albers & Lakens, 2018, for potential drawbacks of using pilot data for sample size planning).

### Reflections on Stimulus Development

We close our discussion by highlighting issues to do with stimulus development that may be of interest to researchers as they adapt experimental instruments with the aim of measuring individual differences in language processing.

The number of trials that contribute to a measure is important for its reliability: the more trials, the better (Rouder & Haaf, 2019; Kuder & Richardson, 1937). When creating linguistic stimuli, there may be a limit to how many “high quality” stimuli we can generate, or participants can reasonably endure. This is especially tricky with constraints such as needing ambiguous words whose meanings are easily depictable. We may need to make compromises and to weigh the costs and benefits of maximising item numbers on the one hand and creating a task that becomes too long and burdensome for participants to complete to their best ability.

As suggested by Holmboe (2022), extensive piloting may enable us to develop item sets that are sensitive to individual differences. With a big enough dataset, item quality can also be formally investigated using psychometric approaches like Item Response Theory (IRT; see Baylor et al., 2011, for a primer on IRT-based psychometrics on language measures). Similarly, cross-validation approaches (e.g., comparing a novel measure with a gold-standard or other existing measures that purport to measure the same construct), provide opportunities to assess the psychometric adequacy of a measure. A new iteration of our task has been developed and is currently being investigated for its psychometric properties, and we hope to make it available for other researchers to use in the future.

## Conclusions

The development of any psycholinguistic task is a careful balancing act. The same experimental design will not be able to simultaneously answer both a group-level research question and one about individual-differences within the same study. We therefore strongly caution against the relatively widespread approach of designing studies to detect group-level effects and then expecting this same dataset to adequately answer important questions about individual differences. An initial group-level study might provide data about the feasibility of a particular task or set of stimuli to detect individual differences. However, as our own case study has demonstrated, measures from group-level multi-version experiments need to be treated as preliminary. Insights from group-level studies are important, especially as they can identify processes of interest that are theoretically motivated. Clearly, it is important to properly understand individual differences in language processing, but this endeavour requires substantial investment to bring about confidence in adequate measurement.

## Data Accessibility Statement

Materials, data and code are available on the OSF, https://osf.io/5z49n/.

## Additional File

The additional file for this article can be found as follows:

10.5334/joc.317.s1 Supplementary Materials. The file contains a detailed description of the methods used to collect narrative naturalness ratings, contextual fit of the target word within the narrative, and word-picture representativeness ratings.
