# Supplementary material for: Studying Individual Differences in Language Comprehension: The Challenges of Item-Level Variability and Well-Matched Control Conditions
Source: J Cogn. 2023 Sep 7;6(1):54. doi: 10.5334/joc.317 (PMC10487189; doi:10.5334/joc.317)
Supplement: Supplementary Materials. — The file contains a detailed description of the methods used to collect narrative naturalness ratings, contextual fit of the target word within the narrative, and word-picture representativeness ratings. [file joc-6-1-317-s1.pdf]

Supplementary Materials

Narrative naturalness ratings

To determine whether our conditions differed in how the narratives felt to native speakers of British English, we ran a pre-test using a rating task. Participants were instructed to read and rate the narratives on the basis of how "natural" each story felt to them, based on whether it "flows well and […] makes sense". The final key words were removed from the narratives so that ratings would not be influenced by whether disambiguation of the final word was required. Narratives were randomly split into two lists of 33 items per condition each to avoid an overly long task. Before each trial, a fixation cross was presented for 500ms (with a 100ms blank screen before and after). The first sentence of the narrative then appeared on the screen. Participants were instructed to read the narratives at their own pace by pressing the 'next' button to proceed to the next sentence. When all three sentences were on the screen (with the final word replaced by '____'), the participants were promptedto rate 'how natural do you find this story as a whole?' on a scale from 1 (Entirely unnatural) to 7 (Perfectly natural). Once they had selected a rating, participants manually moved onto the next trial. Three attention check trials were included in the experiment. In two of the attention trials, the second and third sentences were replaced with 'Are you paying attention?' and 'Click on number X below ____'. In the third attention trial, the first sentence was replaced with 'Click on the number X below to show you are paying attention'. No test sentences were included in these attention tests. Participants were required to get all three answers correct for their data to be included.

Data was collected until we reached 30 participants per list who had passed the attention check (corresponding to 30 data points per item). In total, 66 participants completed the task. 6 were rejected because they did not pass the attention checks, leaving 60 people in

the analysis (41 female). Participants had a mean age of 28.3 (SD=5.9), were majority monolingual, and all spoke British English as their dominant language.

Contextual fit of the target word within the narrative

To determine whether our conditions differed in extent to which the final, target word was predictable fromthe preceding narrative context, we used a measure of contextual fit based on Latent Semantic Analysis (LSA, Landauer & Dumais, 1997). We estimated the semantic distance between the final, target word and the rest of the narrative.

In order to calculate LSA scores for our narratives, the Colorado LSA website ‘pairwise comparison’ function was used (http://lsa.colorado.edu/). Parameters were set according to advice from the LSA Handbook (Dennis, 2014). The topic space was set to ‘general reading up to 1st year college’ and the number of factors was set to 300. The comparison type was set to ‘term to document’ so that the appropriate model weighting was used for comparing the final word to all preceding context. Text was pre-processed such that the final word of each narrative was placed on a separate line to the rest of the narrative. Punctuation was left as it appeared in the original stimuli since this is not included in the LSA calculation. Scores were calculated for all 66 items in each of the three conditions.

Word-picture representativeness rating

To determine whether our conditions differed in the quality of their picture probes, we ran a pre-test using a rating task on the pictures that we had chosen to represent the meanings of the final, target words. The word-picture pairs were presented to participants who were instructed to rate how well the picture represented the word’s meaning on a scale from 1 (“represents the word’s meaning not at all”) to 7 (“represents the word’s meaning perfectly”). Each key word was presented in capital letters in black font on a white background at the top

of the screen, together with a short definition of its (subordinate) meaning in regular typeset underneath. These definitions were included to make sure that participants rated the pictures in relation to the intended meaning of the word. Definitions were taken from the Cambridge English Dictionary (https://dictionary.cambridge.org/dictionary/english/), and shortened to include only essential details. For example, the definition for "bonnet" was "a covering for the head". This ensured that specific wording in the definition did not influence the participants' ratings, beyond directing them to the correct meaning of ambiguous items, and that the same definitions could be used for the matched pairs (e.g., "bonnet" and "hat"). Unambiguous words were also briefly defined.

The associated picture was presented in the centre of the screen below the definition, and participants selected a number on a rating scale below from 1 ("not at all") to 7 ("perfectly"). Trial order was randomised for each participant. We included three attention check trials, in which the target word was replaced with 'attention' and the definition was replaced with an instruction to press a specific number on the scale. Random, unrelated pictures were chosen to appear in these trials. These checks relied on participants reading the definition and not just looking at the target word and picture. Participants were required to get all three answers correct for their data to be included. Two lists were created so that the same picture was never presented twice to the same participant. Data were collected until we reached 30 participants per list, i.e. 30 data points per word-picture pairing. In total, 100 people completed the task and 10 people were excluded for failing the attention checks. The remaining 90 participants (59 female) were spread equally across conditions. They were aged between 19-40 (M=29.8, SD=6.0), were all native speakers of British English and were majority monolingual (bilingual=9 (10%)).
